# Interferon-α acutely impairs whole-brain functional connectivity network architecture – A preliminary study

**DOI:** 10.1016/j.bbi.2015.12.011

**Published:** 2016-11

**Authors:** Ottavia Dipasquale, Ella A. Cooper, Jeremy Tibble, Valerie Voon, Francesca Baglio, Giuseppe Baselli, Mara Cercignani, Neil A. Harrison

**Affiliations:** a Department of Electronics, Information and Bioengineering, Politecnico di Milano, Milan, Italy; b IRCCS, Fondazione don Carlo Gnocchi, Milan, Italy; c Clinical Imaging Sciences Centre, Brighton and Sussex Medical School, Brighton, UK; d Department of Gastroenterology, Brighton & Sussex University Hospitals, Brighton, UK; e Department of Psychiatry, University of Cambridge, Cambridge, UK; f Cambridge and Peterborough NHS Foundation Trust, Cambridge, UK; g Sackler Centre for Consciousness Science, University of Sussex, Falmer, UK; h Sussex Partnership NHS Foundation Trust, Brighton, UK; i Neuroimaging Laboratory, Santa Lucia Foundation, Rome, Italy

**Keywords:** Cytokine, Inflammation, Interferon-α, Resting state fMRI, Graph theory, Functional connectivity, Brain network

## Abstract

Interferon-alpha (IFN-α) is a key mediator of antiviral immune responses used to treat Hepatitis C infection. Though clinically effective, IFN-α rapidly impairs mood, motivation and cognition, effects that can appear indistinguishable from major depression and provide powerful empirical support for the inflammation theory of depression. Though inflammation has been shown to modulate activity within discrete brain regions, how it affects distributed information processing and the architecture of whole brain functional connectivity networks have not previously been investigated.

Here we use a graph theoretic analysis of resting state functional magnetic resonance imaging (rfMRI) to investigate acute effects of systemic interferon-alpha (IFN-α) on whole brain functional connectivity architecture and its relationship to IFN-α-induced mood change. Twenty-two patients with Hepatitis-C infection, initiating IFN-α-based therapy were scanned at baseline and 4 h after their first IFN-α dose. The whole brain network was parcellated into 110 cortical and sub-cortical nodes based on the Oxford-Harvard Atlas and effects assessed on higher-level graph metrics, including node degree, betweenness centrality, global and local efficiency.

IFN-α was associated with a significant reduction in global network connectivity (node degree) (*p* = 0.033) and efficiency (*p* = 0.013), indicating a global reduction of information transfer among the nodes forming the whole brain network. Effects were similar for highly connected (hub) and non-hub nodes, with no effect on betweenness centrality (*p* > 0.1). At a local level, we identified regions with reduced efficiency of information exchange and a sub-network with decreased functional connectivity after IFN-α. Changes in local and particularly global functional connectivity correlated with associated changes in mood measured on the Profile of Mood States (POMS) questionnaire.

IFN-α rapidly induced a profound shift in whole brain network structure, impairing global functional connectivity and the efficiency of parallel information exchange. Correlations with multiple indices of mood change support a role for global changes in brain functional connectivity architecture in coordinated behavioral responses to IFN-α.

## Highlights

• IFN-α induced deep topological changes in whole-brain functional network connectivity.
• Global reduction in nodal connectivity and network efficiency observed within 4 h.
• Local changes observed in a sub-network connecting striatum to cortex.
• Global changes in network efficiency correlated tightly with associated mood change.

## 1 Introduction

Systemic inflammation rapidly impairs mood, motivation and cognition and when chronic is implicated in the etiology of depression (Dantzer et al., 2008). Arguably, the most powerful empirical support for an etiological role for inflammation in depression comes from patients with chronic Hepatitis-C infection treated with interferon-alpha (IFN-α) based therapies. Though clinically efficacious, direct and/or indirect actions of IFN-α on the brain frequently result in highly disabling behavioral changes including fatigue, mood, motivation and cognitive impairments (Capuron et al., 2002). In one third of patients these changes evolve to appear indistinguishable from major depression (Bonaccorso et al., 2001, Dantzer et al., 2008).

Though major depression typically only develops after many weeks of IFN-α administration, changes in mood, motivation and fatigue (and in some cases feelings of social connection and spatial memory) can be readily observed within hours of IFN-α administration (Dowell et al., 2015) and/or other experimental inflammatory challenges such as Typhoid vaccination (Harrison et al., 2009a, Harrison et al., 2009b, Harrison et al., 2014, Harrison et al., 2015a) and Lipopolysaccharide (LPS) injection (Reichenberg et al., 2001, Eisenberger et al., 2010a, Eisenberger et al., 2010b). Furthermore, inflammation also alters physiology, including the central autonomic regulation of the gastrointestinal (Pacheco-Lopez and Bermudez-Rattoni, 2011) and cardiovascular (Harrison et al., 2013) systems. This characteristic profile of inflammation-induced neuropsychological and physiological changes signifies a complex motivational reorientation and suggests that peripheral inflammation can rapidly modify the functional integration of a broad range of interconnected cortical and sub-cortical structures.

To date, rodent and human brain imaging studies have been successful in identifying a discrete set of cortical and sub-cortical structures that appear particularly sensitive to changes in peripheral inflammation. These include the amygdala, striatum (particularly ventral regions), substantia nigra, insula, sub-genual and dorsal anterior cingulate, orbitofrontal cortex and hippocampus/parahippocampus. Some structures appear to play relatively specific roles in discrete aspects of inflammation-associated behavioral change. For example, actions on the ventral striatum (Eisenberger et al., 2010b, Capuron et al., 2012, Harrison et al., 2015b) in impaired reward sensitivity, and hippocampus/parahippocampus in acute spatial memory impairment (Yirmiya and Goshen, 2011, Harrison et al., 2013) whereas other regions such as the insula, anterior and sub-genual cingulate and amygdala appear to play broader less circumscribed roles (Harrison et al., 2009a, Dowell et al., 2015). Common to many of these regions is that they form part of the extended limbic circuitry critical to complex motivational behavior, emotion, learning, and memory and the integration of behavioral and physiological allostatic responses to infection (Critchley and Harrison, 2013, McEwen and Gianaros, 2010).

However, what remains poorly understood is how inflammation modulates brain function at the network level. Broadly, even the simplest cognitive functions depend on the carefully coordinated activity of multiple spatially distributed brain areas. In this context the brain can be viewed as a complex network of nodes (discrete grey matter areas) and inter-connecting fiber pathways. Functional connectivity between nodes can be quantified by acquiring functional MRI (fMRI) data at rest then measuring how activity recorded at each node correlates with that at all other nodes. Conceptualizing the brain in this manner allows the application of advanced mathematical network analyses such as graph theory that can quantify a number of fundamental properties of complex networks. For example, node degree (the number of direct connections to all other network nodes), betweenness centrality (the number of connections between other node pairs that pass through a specific node) and network efficiency (a measure of the networks capacity for parallel information transfer).

Similar to many other complex systems, application of graph theory approaches to the human brain has shown that it follows an efficient ‘small-world’ functional architecture (Achard et al., 2006); i.e. individual network components (nodes) have greater local interconnections (edges) than expected for a random network, and smaller minimum path lengths between node pairs than regular or lattice type networks (Watts and Strogatz, 1998). This functional architecture affords a number of substantial benefits; it reduces wiring cost and ensures a high degree of robustness, i.e. preservation of network integrity following random damage to a node or individual connection (edge). However, such networks also have a smaller number of highly connected (hubs) and ‘high centrality’ nodes that provide the shortest connection path between many other node pairs (high centrality); these nodes are crucial to efficient communication (van den Heuvel et al., 2008) but also vulnerable to targeted insults that can result in a rapid reduction in network efficiency and whole brain connectivity. Whether IFN-α induces rapid, coordinated shifts in behavior through global effects on network efficiency (as may be anticipated from alterations in broadly acting neuromodulators such as dopamine or serotonin) or instead more selective actions on discrete sub-networks or high centrality/node degree regions is currently unknown.

The aim of the present study was therefore to investigate acute effects of IFN-α on the functional connectivity architecture of the human brain with a particular focus on efficiency of information transfer. We used resting state functional magnetic resonance imaging (rfMRI), a powerful technique for investigating human functional brain connectivity (Fox and Raichle, 2007) that enables examination of brain network properties without *a priori* assumptions about regions potentially affected by IFN-α.

**Fig. 1:**
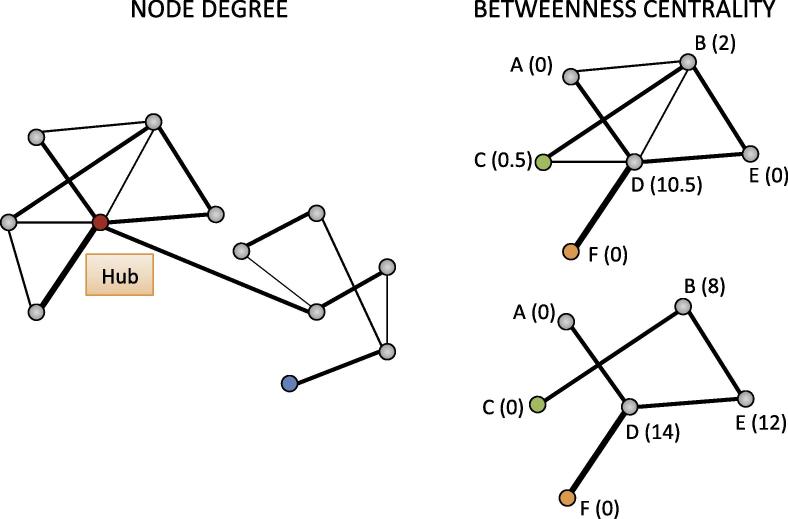
Graphical representation of node degree and betweenness centrality. On the left the red dot (node) represents a high degree node and is directly connected with six out of 11 nodes; the blue dot represents a low degree node and is directly connected with only one node. On the right the upper graph represents a physiological network. Nodes A, B, C, D, E and F are well connected and maintain efficient network communication. Numbers in parentheses refer to each node’s betweenness centrality, which indicates how many of the shortest paths between all other node pairs in the network pass through it. For example, to reach node C (green dot) from node F (orange dot), information flow is efficient and only passes through D. In the lower graph, some connections have been lost. To reach C from F, information now has to go through more nodes (D, E and B). (For interpretation of the references to color in this figure legend, the reader is referred to the web version of this article.)

Twenty-two patients with Hepatitis-C initiating IFN-α-based therapy underwent resting-state fMRI (rfMRI) approximately 1-week before then again 4 h after starting IFN-α-based treatment. rfMRI was parcellated into 110 cortical and sub-cortical regions then higher level graph theory metrics were used to examine effects of IFN-α on topological and weighted properties of the whole-brain network. Specifically, we looked at two complimentary metrics: (1) node degree – a measure of the number of connections (edges) of each node with the other network nodes and (2) betweenness centrality – a measure of how many shortest paths between all other network node pairs pass through any particular node (Bullmore and Sporns, 2009) [see Fig. 1]. We also assessed local and global network efficiency defined as a function of the minimum path length between nodes i.e. whether information can be directly transferred from node A to node B (short path length) or alternately must first pass through one or more intermediary nodes (longer path length). Efficiency thus provides a quantitative measure of the capacity of a network for parallel information transfer between regions. Global efficiency provides a general description of whole-brain network functioning while local efficiency gives an estimate of the importance of each individual node for network information exchange. Functional connectivity changes were also estimated to identify sub-networks particularly sensitive to the acute effects of IFN-α.

We predicted that IFN-α would acutely impair global network functional connectivity, specifically a reduction in node degree and network efficiency. We additionally predicted that highly connected (hub) nodes that make the greatest contribution to global efficiency would be particularly affected. We adopted an exploratory approach to investigate how global or local changes in network function related to acute changes in mood measured on the Profile of Mood States (POMS) questionnaire.

## 2 Materials and methods

### 2.1 Participants

Twenty-two patients (15 male, mean 48.9 ±  11.3 years) initiating IFN-α based therapy for Hepatitis-C infection were recruited. All were fluent in English, aged 18–64 years and fulfilled National Institute for Clinical Excellence (NICE) guidelines for starting pegylated IFN-α based therapy. Participants had a baseline psychiatric evaluation of current mental state and previous psychiatric history, using the MINI International Neuropsychiatric Inventory (MINI) (Sheehan et al., 1998). Participants were excluded if they were receiving treatment for depression at study enrollment, had a history of psychotic illness or autoimmune disease, had not abstained from substance abuse for at least 6 months, were co-infected with HIV or had any cause for liver disease other than HCV. The study was approved by the Cambridge Central National Research Ethics Committee and all participants provided written informed consent.

### 2.2 Study design and behavioral analyses

Participants were evaluated at baseline (mean 7 days before treatment onset) and 4 h after their first IFN-alpha injection. Effects of IFN-α on transient, distinct mood states were assessed using a modified, 36-item version of the Profile of Mood States (POMS) (McNair, 1971). Six items were taken from the vigor, tension-anxiety, depression-dejection, and confusion scales and five items from the fatigue scale of the original POMS with four extra items added to assess symptoms associated with mild infection (fever, aching joints, nausea, and headache) as described previously (Wright et al., 2005). Participants were asked to rate how they felt at that moment on a 5-point scale from 0 = ‘not at all’ to 4 = ‘extremely’. Scores for the five POMS subscales were computed by summing ratings on individual items. Total mood scores were derived by the standard method detailed in the POMS rating manual of subtracting ratings on the negative scales (tension-anxiety, depression-dejection, confusion, and fatigue) from the vigor scores as reported previously (Harrison et al., 2009a).

Magnetic resonance imaging (MRI) followed by blood sampling was repeated at baseline (BASE) and 4 h after the first IFN-α injection (IFN) to index acute effects of IFN-α on brain functional connectivity and circulating cytokines respectively. Of the total cohort 20 (17 male, mean 49.6 ± 11.2 years) completed both blood samples. Demographic and cytokine data are reported in Table 1. All participants completed both MRI sessions.

Psychopathological symptoms were additionally evaluated at each visit as well as 4, 8, 12 and 24 weeks of IFN-α based therapy using the Epworth Sleepiness Scale (ESS) (Johns, 1991), fatigue Visual Analogue Scale (fVAS), Hamilton Depression Rating Scale (HAMD), State and Trait Anxiety Inventory (STAI) and MINI. These data form part of a larger study and are not reported here.

### 2.3 Image acquisition

MR imaging was performed on a 1.5T Siemens Avanto (Siemens AG Medical Solutions, Erlangen, Germany) equipped with a 32-channel head-coil. Functional MRI data were obtained during rest using a T2^∗^-weighted EPI sequence (TR = 2520 ms; TE = 43 ms; flip angle = 90°; resolution = 3 × 3 × 3 mm, with 20% between-slice gap; matrix size = 64 × 64; 34 axial slices; 190 volumes). A 3D T1-weighted anatomical scan was obtained for each participant in one session using an MPRAGE acquisition (TR = 2730 ms, TE = 3.57 ms, TI = 1000 ms, flip angle = 7°). Task-based fMRI was additionally acquired and will be reported separately.

### 2.4 Image analysis

Pre-processing of resting state fMRI (rsfMRI) data was performed using FSL (Jenkinson et al., 2012, Smith et al., 2004). Standard pre-processing steps involved: motion correction, removal of non-brain tissue, spatial smoothing with a 5 mm full width at half maximum Gaussian kernel and high-pass temporal filtering with a cut-off frequency of 0.01 Hz. Single-subject spatial independent components analysis (ICA) with automatic dimensionality estimation was subsequently performed using Multivariate Exploratory Linear Optimized Decomposition into Independent Components (MELODIC, Beckmann and Smith, 2004). The ICA-based Xnoiseifier (FIX, Salimi-Khorshidi et al., 2014) was used to regress the full space of motion artifacts and noise components out of the data (Griffanti et al., 2014). The FIX training dataset used to discriminate Blood Oxygen Level Dependent (BOLD) components and artifact was built using participants’ baseline data. After pre-processing, each subject-specific 4D dataset was aligned to their corresponding MPRAGE structural image using FMRIB’s Linear Image Registration Tool (FLIRT) (Jenkinson and Smith, 2001, Jenkinson et al., 2002) enhanced with brain-boundary registration (Greve and Fischl, 2009). rsfMRI datasets were then normalized to MNI152 standard space using FMRIB’s Non-linear Image Registration Tool (FNIRT) (Andersson et al., 2010) and simultaneously resampled to 2 × 2 × 2 mm resolution.

### 2.5 Regions of interest

rsfMRI were parcellated into 96 cortical and 14 sub-cortical areas using the Harvard-Oxford atlas (Makris et al., 2006, Frazier et al., 2005, Desikan et al., 2006, Goldstein et al., 2007) then time-series extracted from each of these 110 anatomical regions of interest.

### 2.6 Wavelet-correlation matrices

Maximum overlap discrete wavelet transforms (MODWT, Percival and Walden, 2000) were used to decompose the 110 time series into wavelet coefficients at four scales determined by the MODWT algorithm (scale 1: 0.03–0.099 Hz; scale 2: 0.0165-0–03 Hz; scale 3: 0.007–0.0165 Hz; scale 4: range 0.003–0.007 Hz) and wavelet-correlation matrices estimated at each scale (Bullmore et al., 2004, Achard et al., 2006, Achard and Bullmore, 2007). Resting state BOLD signal frequencies are predominantly located in the 0.012–0.1 Hz frequency band with lower frequencies predominated by non-physiological sources (Fransson, 2005). We therefore restricted our analyses to the two higher frequency scales (scale 1 and scale 2). Of note, data in the two lower frequency scales predominated by physiological noise had also been largely removed by our earlier high-pass filtering at 0.01 Hz. Wavelet correlation matrices were converted into *z*-values and averaged for all patients before and after IFN-α administration.

### 2.7 Graph analysis and functional connectivity

Higher-level graph metrics, including node degree, betweenness centrality, global and local efficiency, were estimated using the Brain Connectivity Toolbox (Rubinov and Sporns, 2010). Details of graph theory and its application to brain networks can be found elsewhere (Bullmore and Sporns, 2009), together with a full description of many of the indices that can be derived from it. For the purposes of the current investigation, we focused on 2 high-level indices: node degree and betweenness centrality. Node degree describes the number of connections of each node. Nodes with high degree (many connections) are often termed “hubs” and are believed to make a greater contribution to global network efficiency than less well connected nodes. Betweenness centrality is defined as the fraction of all the shortest paths between nodes that pass through a given node. For example, a high centrality node would provide the shortest connection path between many other pairs of nodes (see Fig. 1).

We predicted that IFN-α would acutely impair global network functional connectivity, resulting in a reduction in mean node degree and increase in betweenness centrality. Global node degree and betweenness centrality were evaluated on unweighted (i.e. binarized) graphs. Brain functional networks have been shown to express economical small-world properties (Watts and Strogatz, 1998, Latora and Marchiori, 2001, Achard et al., 2006), we therefore thresholded our functional connectivity matrices with low *K* values that preserve only the strongest functional connections and support highly efficient parallel information processing at a relatively low wiring cost (Latora and Marchiori, 2001). Graph theoretic analyses can also be sensitive to threshold value. To investigate the robustness of our findings we therefore report our global network analyses at four separate cost *K* thresholds within the small-worldness range (0.05 ⩽ *K* ⩽ 0.35 with increments of 0.1), where *K* represents the actual number of edges (connections) in the graph estimated as a proportion of the total number of possible edges (Achard and Bullmore, 2007). All values of betweenness centrality were normalized to the maximum number of shortest paths that any nodes can participate in (i.e. (*N* − 1)*(*N* − 2) for a whole-brain network with *N* = 110 nodes).

Repeated measures ANOVA with factors: Inflammation (Baseline, IFN-α) and *K* value (0.05, 0.15, 0.25, 0.35) were used to investigate effects of IFN-α on node degree and betweenness centrality for the two higher frequency scales. To investigate whether actions of IFN-α selectively impacted high or low node degree/betweenness centrality we repeated this analysis after splitting the distribution of node degree/betweenness centrality data into quintiles. Greenhouse Geisser correction of degrees of freedom was used where appropriate. Node degree and betweenness centrality were also used as local indices to evaluate effect of IFN-α on each individual node and results at BASE and IFN compared using paired sample *t*-tests.

Global and local efficiency was estimated using weighted (i.e. non-binarized) graphs that incorporate information on the strength of nodal connections (Bullmore and Sporns, 2009). Local efficiency was used to measure effects of IFN-α on each discrete region (node). Global efficiency (mean local efficiency across nodes) indexed effects on global network connectivity. Analyses of the distribution of node degree and betweenness centrality showed that scale 2 findings were robust to different *K* values. We therefore restricted further analyses to the scale 2 weighted graphs by thresholding the wavelet-correlation matrices at the conservative cost *K* = 0.25. This approach enabled us to minimize the number of spurious edges in each network while preserving information about the connection weights between nodes. Significant global and local efficiency changes induced by IFN-α (BASE and IFN) were then evaluated using paired sample *t*-tests.

Scale 2 wavelet-correlation matrices thresholded with *K* = 0.25 were then used in the Network Based Statistic toolbox (NBS) (Zalesky et al., 2010) to identify actions of IFN-α on the functional connectivity of specific network sub-components. Specifically, a paired *t*-test was performed to test for a between-condition difference in the correlation coefficient (expressed as *z*-score) at each of the 5995 (=N*(*N* − 1)/2, with *N* = 110 nodes) pairs of regions. Specific graph sub-components were then identified among the connections with a *t*-statistic exceeding a corrected threshold of *t* = 3.5. The NBS was specifically developed to perform Statistics at network level and correct for multiple comparisons while taking into account inter-connections between nodes. A whole network family-wise error (FWE)-corrected *p*-value was calculated for the size of each resulting component using permutation testing (10,000 permutations). The two alternative hypotheses (BASE > IFN and IFN > BASE) were evaluated independently.

For each subject, local efficiency values of those nodes belonging to the sub-network identified with NBS were averaged and the outcomes used to examine effects of IFN-α on sub-network efficiency. One-tailed paired *t*-test was used to assess reductions in network efficiency following IFN-α (BASE > IFN).

## 3 Correlation between rfMRI results and behavioral scales

Finally we investigated whether global and local connectivity measures sensitive to acute IFN-α also correlated with individual’s sensitivity to the behaviorally impairing effects of IFN-α, i.e. changes in POMS subscales of vigor, tension-anxiety, confusion, fatigue, negative and total mood score. The false discovery rate (FDR) was used to correct for multiple comparisons.

## 4 Results

### 4.1 Wavelet-correlation matrices

**Fig. 2:**
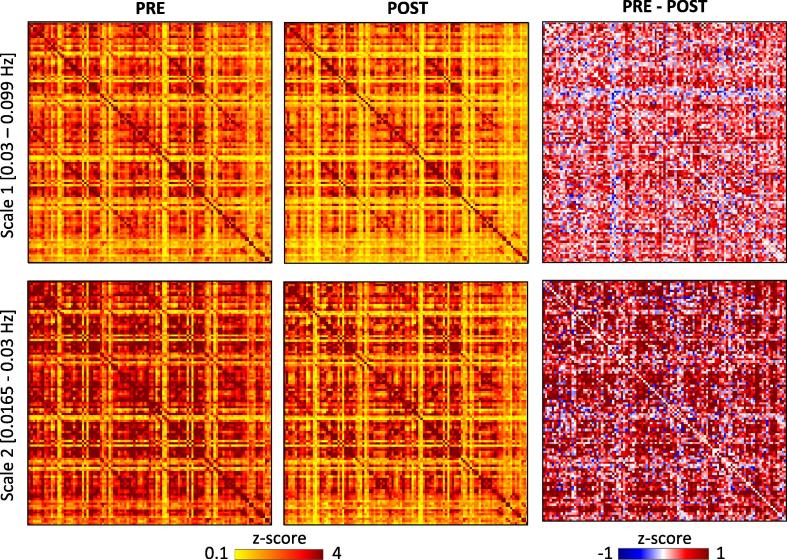
Functional connectivity matrices for frequency ranges 0.03–0.099 Hz (scale 1) and 0.0165–0.03 Hz (scale 2), averaged across all participants before (PRE, first column) and 4 h after IFN-α (POST, second column). Color denotes *Z* score. For both scales, the difference between the two conditions is represented in the third column (PRE minus POST). Red and blue colors respectively indicate higher or lower functional connectivity at baseline than 4 h after IFN-α injection. The difference between PRE and POST is particularly evident for scale 2, as indicated by the more “yellow” colors (indicating lower values) at POST, and by the dark red colors in the difference matrix, indicating difference in *z*-scores around 1 or higher. (For interpretation of the references to color in this figure legend, the reader is referred to the web version of this article.)

Wavelet-correlation matrices at both scale 1 (0.03–0.099 Hz) and scale 2 (0.0165-0–03 Hz), where resting state BOLD signal frequencies predominate, revealed a striking and wide-ranging reduction in functional connectivity within 4-h of starting IFN-α (Fig. 2).

### 4.2 Global effect of IFN-α

**Fig. 3:**
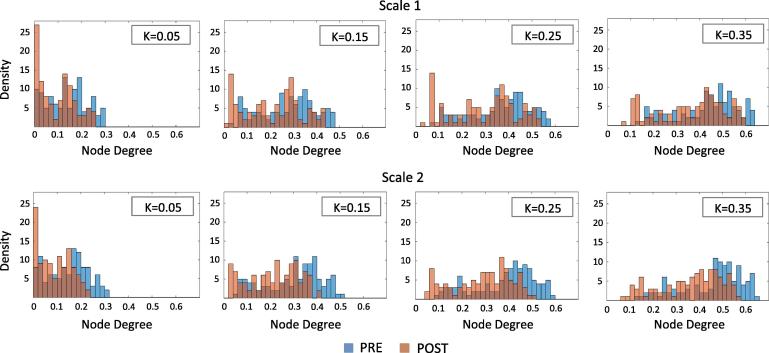
Distribution of mean node degree at baseline (blue) and after IFN-α administration (orange) for cost *K* equal to 0.05, 0.15, 0.25 and 0.35. Node degree histograms are normalized to 109 (the maximum number of possible connections per node). (For interpretation of the references to color in this figure legend, the reader is referred to the web version of this article.)

To investigate this further we next examined effects of IFN-α on the node degree distribution (the number of direct connections of each node) across the whole brain network (Fig. 3). As anticipated, a progressive loss of node degree (identifiable as a shift of the curve to the left) was observed as *K* value was decreased across both conditions for both scale 1 and scale 2. However, more importantly, we also observed a left shift in the node degree distribution following IFN-α that was most prominent for scale 2. To ensure the robustness of this observation we first investigated effects of IFN-α on mean nodal degree across all *K* values (repeated measures ANOVA, factors: Intervention (pre, post-IFN) and *K* value (0.05, 0.15, 0.25, 0.35)). This showed a significant main effect of IFN-α for scale 2 (*F*_(1,21)_ = 5.20, *p* = 0.033) but not scale 1 (*F*_(1,21)_ = 2.08, *p* = 0.164). Importantly, we observed no significant intervention by *K* value interaction (*F*_(1,21)_ = 0.985, *p* = 0.337, *F*_(1,21)_ = 3.64, *p* = 0.068) for either scale confirming that effects of IFN-α on mean node degree were robust to different threshold (*K*) values. Further, effects of IFN-α on scale 2 mean nodal degree were consistent across *K* values (*p* = 0.030–0.039).

In contrast to effects on node degree, IFN-α appeared to induce a right shift in betweenness centrality (the number of shortest paths between nodes that pass through each individual node) (data not shown). However, analysis of effects of IFN-α on mean betweenness centrality across all *K* values (repeated measures ANOVA, factors: Intervention (pre, post-IFN) and *K* value (0.05, 0.15, 0.25, 0.35)) revealed no significant main effect of IFN-α at either scale (Scale1: *F*_(1,21)_ = 0.298, scale 2: *p* = 0.591, *F*_(1,21)_ = 1.494, *p* = 0.235).

Finally, we assessed effects of IFN on global efficiency scores (a measure of capacity for parallel information transfer). We observed a significant reduction in global efficiency after IFN-α at scale 2 (*t*_(21)_ = 2.38, *p* = 0.013). Effects on scale 1 showed a similar trend but did not reach statistical significance (*t*_(21)_ = 1.55, *p* = 0.068).

### 4.3 Effect of IFN-α as a function of node centrality

To investigate whether the effects of IFN-α were greater for more highly connected ‘hub’ nodes, we collapsed the data across *K* value and split the node-degree distribution into quintiles. We then repeated the scale 2 analysis using node quintile and intervention (pre, post-IFN) as within subject factors. This showed the expected main effect of quintile on node degree (*F*_(1,21)_ = 377.14, *p* < 0.001), however there was no significant IFN-α by quintile interaction confirming that effects of IFN-α of node degree were not significantly different for high versus low degree nodes.

Performing a similar analysis after splitting the betweenness centrality scores into quintiles showed no significant main effect of node quintile (*F*_(1,21)_ = 0.297, *p* = 0.592, *F*_(1,21)_ = 1.484, *p* = 0.237) and no significant IFN-α by quintile interaction for either scale (Scale 1: *F*_(1,21)_ = 0.589, *p* = 0.458, scale 2: *F*_(1,21)_ = 2.161, *p* = 0.155) confirming that IFN-α has no significant effect on betweenness centrality.

**Fig. 4:**
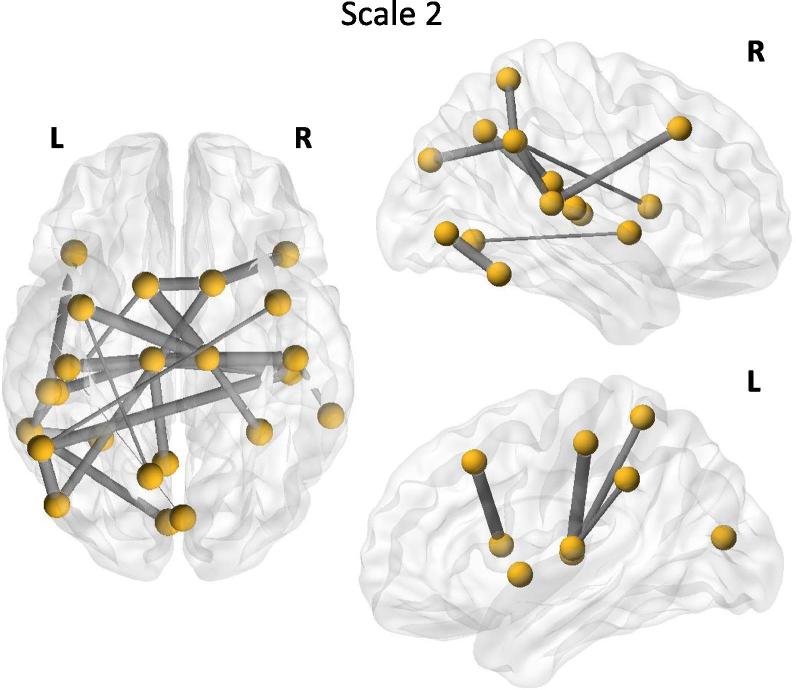
Graphical representation of the scale 2 sub-network showing a significant reduction in functional connectivity 4-h after IFN-α. Thickness of edges (lines) is proportional to the magnitude of IFN-α induced reductions in functional connectivity.

*Local effects of IFN-α*: We next investigated whether actions of IFN-α localized to a specific functional sub-network or discrete nodes using the network based statistic method to correct for multiple comparisons (*p*_corr_ < 0.05) (Fig. 4). This identified effects of IFN-α on a single cortical-subcortical network that included a number of regions e.g. bilateral insula, frontal cortex and caudate, previously implicated in inflammation-induced mood, motivation and cognitive change (Table 2). Repeating our global efficiency analysis on this sub-network again confirmed a significant reduction in global (sub-network) efficiency within 4 h of IFN-α administration (*t*_(21)_ = 2.54, *p* = 0.0096). Analysis of node degree additionally identified three left sided regions: nucleus accumbens, thalamus and inferior temporal gyrus that showed the most significant reduction in node degree post IFN-α and that survived whole brain FDR correction (Table 3).

### 4.4 Relationship between network connectivity changes and sickness symptoms

We investigated whether IFN-α-induced changes in global and regional network connectivity also related to individual sensitivity to the behaviorally impairing effects of IFN-α within the four POMS domains of vigor, tension-anxiety, confusion and fatigue as well as the POMS negative and total mood scores. Significant IFN-α induced changes in fatigue (*t*_(21)_ = 2.06; *p* = 0.026) and confusion (*t*_(21)_ = 1.98; *p* = 0.03) were observed.

Interestingly however, changes in Global (whole brain) network efficiency showed strong negative correlations with 5/6 of these POMS measures (not vigor) (Table 4). Four of these measures (not vigor or total mood) also correlated with sub-network efficiency and three (not vigor, total mood or confusion) with IFN-α induced changes in left ventral striatal and thalamic nodal. Ten of these 15 correlations also survived FDR correction for multiple comparisons. Together, supporting the suggestion that IFN-α induced changes in functional brain connectivity networks underpin acute motivational reorientation associated with IFN-α.

## 5 Discussion

Here, using a graph theoretic analysis of resting state fMRI data, we show that peripheral IFN-α rapidly distorts whole brain functional network architecture, mean nodal connections are globally diminished, global capacity for parallel information transfer impaired and efficiency of a discrete cortical-subcortical sub-network reduced. Furthermore, many of these network/sub-network level changes showed good correlations with concomitant mood and cognitive change, suggesting that IFN-α may rapidly induce coordinated shifts in behavior through actions at the level of network organization.

To date, studies investigating the neurobiology of human inflammation-induced behavioral change have provided important insights into the set of cortical and sub-cortical structures that underpin different aspects of behavioral change. For example, actions on ventral striatum have been repeatedly implicated in inflammation-induced reductions in reward sensitivity (Eisenberger et al., 2010b, Capuron et al., 2012, Harrison et al., 2015b), substantia nigra in psychomotor slowing and motivational responses to novelty (Brydon et al., 2008, Harrison et al., 2014) and amygdala, insula, sub-genual and dorsal anterior cingulate and dorso-lateral and orbitofrontal cortex to effects on mood and emotional reactivity (Harrison et al., 2009a), pain sensitivity (Benson et al., 2015), cognitive slowing (Harrison et al., 2009b) and autonomic reactivity (Harrison et al., 2013). These studies illustrate the spatially distributed nature of effects of inflammation on the brain and highlight the potential importance of broadly acting neuromodulators like dopamine and serotonin. However few studies have investigated how inflammation impacts on distributed information processing and the architecture of whole-brain functional connectivity networks.

Within the broader neuroimaging field, recognition that even the simplest cognitive functions involve highly distributed processing has led to a rapid shift away from attempts to map higher cognitive functions to discrete brain regions towards relating them to broader changes in functional brain connectivity networks (Johansen-Berg, 2013). This conceptual shift has already illustrated the power of network-based analyses to further our understanding of a number of biologically important processes. For example, graph theoretic measures of global network efficiency have been shown to underscore IQ differences in healthy adults (Li et al., 2009), while Alzheimer’s disease has been shown to selectively target critical high node degree ‘hub’ regions that interconnect distinct, functionally specialized systems (Buckner et al., 2009). Our current findings are the first to demonstrate that peripherally administered IFN-α rapidly impairs global network efficiency and mean node degree, effects that were directly associated with associated impairments in mood and cognition.

Of all the topological features that can be estimated by graph theory, node degree, which measures the number of connections that link each node to the broader network, is one of the most fundamental. It is therefore noteworthy, that in accord with our prior hypothesis, IFN-α resulted in a rapid reduction in mean global node degree i.e. the mean number of network connections (edges). Importantly, this finding, performed on binarized, unweighted graphs, was robust across a range of physiologically plausible cost thresholds (*K*). However, against our prior prediction, this effect was uniform across nodes, rather than being selective for high node degree (hub) regions. Thus unlike the situation observed in Alzheimer’s disease where critical high connectivity hub regions appear selectively targeted, actions of IFN-α appear to have a more global impact on network connectivity. The robustness of this finding was further supported by our finding of a significant reduction in global network efficiency (a measure of parallel information processing) as well as the lack of evidence for any effect of IFN-α on the distribution of betweenness centrality (an index of a node’s centrality within the whole-brain network) at any scale or cost value *K*.

The mechanistic basis for how peripherally administered IFN-α can so rapidly impair global network connectivity is currently uncertain. However, our observation that these actions are effected on a global scale points towards a likely role for neuromodulators such as dopamine, norepinephrine or serotonin that can rapidly alter diverse and widespread neuronal populations rather than a more regionally targeted effect. In support of this interpretation, inflammation has been linked to altered nucleus accumbens dopamine efflux in rodents (Borowski et al., 1998), decreased striatal dopamine release in rhesus monkeys (Felger et al., 2013, Felger et al., 2015) and reduced presynaptic dopamine synthesis or release in humans (Capuron et al., 2012). Further, monkeys showing behavioral impairment after inflammatory challenge with lipopolysaccharide exhibit significantly lower cerebrospinal fluid concentrations of the dopamine metabolite homovanillic acid (Felger et al., 2007).

More broadly, peripheral IFN-α has also been shown to decrease the neural availability of tetrahydrobiopterin, an enzyme co-factor that is essential to the biosynthesis of all monoaminergic neurotransmitters (Kitagami et al., 2003). This process appears dependent on nitric oxide (NO), a small molecule that readily crosses the blood–brain-barrier and whose synthesis is induced by IFN-α (Kitagami et al., 2003). Inflammatory cytokines can also impair 5-hydroxytryptamine (5-HT) synthesis by inducing indoleamine 2,3-dioxygenase (Dantzer et al., 2008) and reduce synaptic monoamines by enhancing the function of monoamine reuptake transporters (Krishnadas et al., 2016, Kamata et al., 2000). Together, these mechanisms illustrate how pro-inflammatory cytokines can induce widespread reductions in brain monoamine neurotransmitters and provide a potential mechanistic link to the global reductions in network connectivity we observed.

Complimenting our global network analyses we also used a network based statistics approach to investigate whether specific sub-networks or discrete network nodes showed particular sensitivity to IFN-α. These analyses identified three left sided regions: nucleus accumbens, thalamus and temporo-occipital division of the inferior temporal gyrus that showed a statistically significant (whole network corrected) reduction in network connections (node degree) following IFN-α. This finding is noteworthy as it supports earlier studies showing bilateral (though left predominant) increases in striatal ^18^fluoro-deoxy-glucose (Capuron et al., 2007) and ^18^fluorodopa uptake (Capuron et al., 2012) as well as left-sided increases in striatal glutamate/creatine ratio (Haroon et al., 2014) and magnetization transfer (Dowell et al., 2015) following IFN-α. Bilateral caudate and thalamic connections to frontal, parietal, temporal and insula nodes also formed part of our sub-network that showed particular sensitivity to IFN-α.

The functional significance of these findings was further supported by our findings of significant correlations between changes in global and local network efficiency as well as localized changes in left ventral striatal and thalamic connectivity (node degree) and IFN-α-induced mood change. In other words, participants who reported the greatest impairment in mood following IFN-α also showed the greatest reductions in network connectivity. Interestingly, this effect was most striking for changes in global efficiency, a measure of whole network information processing. Overall, changes in global efficiency showed significant correlations with five of the six POMS sub-scales including total mood score and cognitive confusion. Correlations with global efficiency were also stronger than for any of the measures of local network function, reaffirming the importance of global network changes to the behaviorally impairing effects of IFN-α.

This study has a number of limitations. First, we focused only on acute effects of IFN-α on functional brain network architecture. It is possible that the acute changes we observed will evolve or resolve during chronic IFN-α exposure as a consequence of neuroadaptation to chronic IFN-α exposure. Second, the study was performed in a modest sample size on a 1.5T scanner. Though we adopted an efficient repeated-measure within-subject study design it would be important for future studies to follow-up these findings in larger populations. Finally, while graph theory offers a powerful framework for characterizing networks, its application to brain data is still in its infancy and relies on a number of assumptions and choices. For example, the criteria used for brain parcellation that defines the number of nodes of the graph and thresholds used for identifying edges (connections) can markedly influence the results. We have attempted to minimize the impact of these variables firstly by using the Harvard-Oxford Atlas for node-parcellation and secondly by analyzing our global metrics across the range of plausible values for network cost (*K*). The Harvard-Oxford Atlas is commonly utilized for graph theory analyses (Meng et al., 2014) enabling comparison of our data with other studies using a graph theory methodology. The use of a range of *K* values avoided the risks associated with selection of an arbitrary cost threshold and demonstrated that our findings were robust across the range of plausible cost thresholds.

To conclude, IFN-α rapidly induced a profound shift in whole brain network structure, impairing global functional connectivity and the efficiency of parallel information exchange. These changes in network function were rapid, occurring within 4 h of IFN-α administration and robust across a broad range of physiologically plausible thresholds. Correlations with individual sensitivity to the mood impairing effects of IFN-α support a role for changes in network function. The rapid onset and widespread nature of these effects suggests mediation via actions on monoaminergic neuromodulators such as dopamine or 5-HT, a hypothesis that will need to be tested in future studies.

## Figures and Tables

**Table 1:** Demographic and cytokine data of patients with Hepatitis C.

	Subjects at baseline (mean ± SE)	Subjects 4 h after the first IFN-α injection (mean ± SE)
Number of participants	22	
Age (yrs)	48.9 ± 2.41	
Gender (M/F)	15/7	
IL6 (pg/ml)	1.98 ± 0.46	4.39 ± 0.68
IL10 (pg/ml)	0.84 ± 0.23	1.13 ± 0.25
TNF (pg/ml)	1.88 ± 0.23	2.06 ± 0.24
IFN-α (pg/ml)	3.21 ± 0.92	44.95 ± 7.43
IL1Ra (pg/ml)	504.2 ± 69.40	3705.7 ± 916.80

**Table 2:** Cortical-subcortical regions belonging to the network with reduced functional connectivity post IFN-α at cost *K* = 0.25 (Scale 2).

ID	Left hemisphere	Right hemisphere
0	Frontal pole	
1	Insular cortex	Insular cortex
3	Middle frontal gyrus	Middle frontal gyrus
15	Inferior temporal gyrus, t.p.	
16		Postcentral gyrus
17	Superior parietal lobule	Superior parietal lobule
19	Supramarginal gyrus, p.d.	Supramarginal gyrus, p.d.
22	Lateral occipital cortex, i.d.	
30	Precuneus cortex	
31	Cuneal cortex	
35	Lingual gyrus	
42	Parietal operculum cortex	
44	Heschl’s gyrus	Heschl’s gyrus
45	Planum temporale	
46		Supracalcarine cortex
	Thalamus	Thalamus
	Caudate	Caudate

t.p. = temporo-occipital section; p.d. = posterior division; i.d. = anterior division. ID refers to the Harvard-Oxford atlas classification of the cortical areas (http://neuro.imm.dtu.dk/wiki/Harvard-Oxford_Atlas).

**Table 3:** Regions with reduced node degree post IFN-α at cost *K* = 0.25.

ID	Anatomical Region	*t*_(21)_	*p*
15	Left inferior temporal gyrus, t.p.	3.70	0.0007
–	Left thalamus	3.51	0.0010
–	Left accumbens	3.71	0.0007

ID refers to the Harvard-Oxford atlas classification of the cortical areas (http://neuro.imm.dtu.dk/wiki/Harvard-Oxford_Atlas). t.p. = temporo-occipital section.

**Table 4:** Functional brain network alterations significantly correlated with symptoms (IFN – BASE).

ΔPOMS (IFN – BASE)	Correlation with Δ (IFN – BASE):	*r*	*p*-Value
Tension/anxiety	Global efficiency	−0.59	0.002⁎
	Network efficiency	−0.58	0.002⁎
	ND, left thalamus	−0.51	0.007⁎
	ND, left accumbens	−0.49	0.009⁎

Fatigue	Global efficiency	−0.52	0.007⁎
	Network efficiency	−0.42	0.026
	ND, left thalamus	−0.38	0.040
	ND, left accumbens	−0.47	0.013⁎

Confusion	Global efficiency	−0.37	0.044
	Network efficiency	−0.36	0.050

Negative mood	Global efficiency	−0.65	0.0005⁎
	Network efficiency	−0.64	0.0006⁎
	ND, left thalamus	−0.52	0.006⁎
	ND, left accumbens	−0.56	0.003⁎

Global Mood score^	Global efficiency	0.41	0.030

ND = node degree; t.p. = temporo-occipital part; a.d. = anterior division.

⁎ Correlations survived FDR correction for multiple comparisons.

^ Global mood score decreases with improving mood.
